# A novel hydroxyapatite film coated with ionic silver via inositol hexaphosphate chelation prevents implant-associated infection

**DOI:** 10.1038/srep23238

**Published:** 2016-03-17

**Authors:** Haruki Funao, Shigenori Nagai, Aya Sasaki, Tomoyuki Hoshikawa, Takashi Tsuji, Yasunori Okada, Shigeo Koyasu, Yoshiaki Toyama, Masaya Nakamura, Mamoru Aizawa, Morio Matsumoto, Ken Ishii

**Affiliations:** 1 Department of Orthopaedic Surgery, Keio University School of Medicine, Shinjuku, Tokyo, JAPAN; 2 Department of Microbiology and Immunology, Keio University School of Medicine, Shinjuku, Tokyo, JAPAN; 3 Department of Pathology, Keio University School of Medicine, Shinjuku, Tokyo, JAPAN; 4 Department of Applied Chemistry, School of Science and Technology, Meiji University, Kawasaki, Kanagawa, JAPAN; 5 Department of Molecular Immunology, Graduate School of Medical and Dental Sciences, Tokyo Medical and Dental University, Bunkyo, Tokyo, JAPAN; 6 Department of Pathology and Oncology, School of Medicine, Juntendo University, Bunkyo, Tokyo, JAPAN; 7 Laboratory for Immune Cell Systems, RIKEN Centre for Integrative Medical Sciences (IMS), Yokohama, Kanagawa, JAPAN

## Abstract

Various silver-coated implants have been developed to prevent implant-associated infections, and have shown dramatic effects *in vitro*. However, the *in vivo* results have been inconsistent. Recent *in vitro* studies showed that silver exerts antibacterial activity by mediating the generation of reactive oxygen species in the presence of oxygen. To maintain its antibacterial activity *in vivo*, the silver should remain in an ionic state and be stably bound to the implant surface. Here, we developed a novel bacteria-resistant hydroxyapatite film in which ionic silver is immobilized via inositol hexaphosphate chelation using a low-heat immersion process. This bacteria-resistant coating demonstrated significant antibacterial activity both *in vitro* and *in vivo*. In a murine bioluminescent osteomyelitis model, no bacteria were detectable 21 days after inoculation with *S. aureus* and placement of this implant. Serum interleukin-6 was elevated in the acute phase in this model, but it was significantly lower in the ionic-silver group than the control group on day 2. Serum C-reactive protein remained significantly higher in the control group than the ionic-silver group on day 14. Because this coating is produced by a low-heat immersion process, it can be applied to complex structures of various materials, to provide significant protection against implant-associated infections.

Infections associated with medical devices such as catheters, heart valves, and orthopaedic implants can create serious complications in patients. The pathogenesis of these infections involves the formation of a biofilm^1^. While some implant-associated infections can be resolved by simply removing the foreign body, as with infected catheters, removing critical cardiac or orthopaedic implants is more problematic.

As the use of fracture fixation plates, artificial joints, spinal implants, and similar devices has increased, so has implant-associated osteomyelitis, which causes progressive inflammation and destructive bone changes. Treatment often requires prolonged antibiotic therapy or revision surgeries, which can have long-term health effects. Intravenous antibiotic agents have limited effects against implant-associated osteomyelitis, due to bone’s naturally poor blood supply and to biofilm formation on the implant. Therefore, antimicrobial surface modifications (e.g., antibiotics^2^, quaternary ammonium compounds^3^, iodine^4^, and silver coatings^5^) on implants have been developed to prevent bacteria from adhering and forming a biofilm.

Silver is widely used in wound dressings and medical devices because it exerts broad-spectrum antimicrobial activity against gram-positive and -negative bacteria, viruses, fungi, and protozoa^6^,^7^. Silver ions are reported to bind to membranes, enzymes, and nucleic acids, and to inhibit microorganisms’ respiratory chain^8^,^9^. Silver has low toxicity and is unlikely to provoke microbial resistance. Although various silver-coated implants have been developed to prevent implant-associated infections, and have shown dramatic effects *in vitro*^6^,^10^,^11^, the *in vivo* results have been inconsistent^12^,^13^,^14^. A recent *in vitro* study suggested that silver exerts its antibacterial effects by mediating the generation of reactive oxygen species in the presence of oxygen^15^. However silver’s antibacterial activity is exerted in its ionic state *in vivo*^16^. Thus, devices with good *in vitro* antibacterial effects do not necessarily elicit the same results at an infection site *in vivo*. Moreover, in environments containing albumen, free silver ions precipitate and drop below an effective concentration^6^. In fact, clinical trials have found no significant differences between silver-coated and uncoated urinary catheters, central venous catheters, and heart valves^12^,^13^,^14^. To maintain its antibacterial activity *in vivo*, the silver must remain in an ionic state and be stably bound to the implant surface.

Hydroxyapatite (HAp) is an osteoconductive and biocompatible material that contributes to the main component of bone. Techniques to synthesize silver-coated or silver-containing HAp include the sol-gel procedure^17^, the wet chemical method^18^, ion exchange^19^, and thermal spraying^11^. (Ca_10_(PO_4_)_6_(OH)_2_) film via chelation with an inositol hexaphosphate (IP6) (C_6_H_6_(OPO_3_H_2_)_6_) film. This immersion treatment does not require high-temperature processing.

## Results

**Figure 1:**
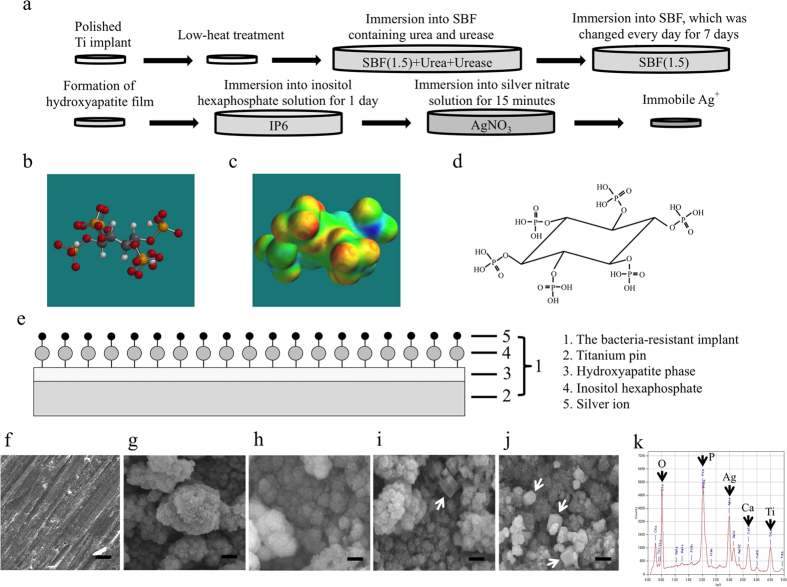
Fabrication of a titanium implant coated with ionic silver bound to a hydroxyapatite (HAp) film via inositol hexaphosphate (IP6)-film chelation, and its surface characteristics. (**a**) The fabrication process involves i) forming a HAp film on a Ti implant, ii) modifying the surface of the HAp film with IP6, and iii) fixing Ag^+^ on the IP6-modified surface of the HAp film by chelation. (**b**) Molecular model of IP6 drawn by Spartan^®^ (Wavefunction, Inc.; white: H, grey: C, red: O, Orange: P). (**c**) Image of the electrostatic potential of model (**b**); red indicates the presence of electrons at high density. (**d**) Structural formula of IP6 by Barré and Courtois; twelve OH groups in the IP6 form chelate-bonding sites for metal ions (ex, Ag^+^, Ca^2+^, Zn^2+^). (**e**) Diagram showing the coating of a Ti implant with HAp, the modification of the HAp surface with IP6, and the application of a layer of immobile ionic silver via IP6’s chelate-binding ability (HAp-IP6-Ag^+^ Ti). (**f**,**g**) Scanning electron microscopy (SEM) images of (**f**) the pure Ti substrate, and (**g**) HAp particles precipitated on the Ti substrate to form a layer. (**h–j**) SEM images of HAp-IP6-Ag^+^(1, 5, 10)-Ti pins, respectively. (**i**,**j**) Arrows indicate cube-shaped particles (arrows) deposited on the HAp layer. Bars = 1 m. (**k**) Energy dispersive X-ray (EDX) spectrum showing the presence of Ag^+^ on a HAp-IP6-Ag^+^(10)-Ti pin.

Here we fabricated a titanium (Ti) implant coated with ionic silver by i) forming a HAp film on the implant, ii) modifying the HAp-film surface with IP6, and iii) immobilizing silver ions (Ag^+^) on the modified film by IP6 chelation (Fig. 1a). IP6 is a biocompatible component that exists in plants as myoinositol hexakisphosphate^20^; it strongly chelates minerals and some metal ions^21^. Figure 1b–d shows the chemical structure of IP6. We previously used IP6 as a chelating agent to form an IP6-HAp cement^22^.

In detail, we used commercially available pure Ti pins (0.5 mm diameter x 8 mm length, Nilaco Co., Japan) as the implants, heated them to 200 °C, and immersed them in a surface-treatment (ST) solution, as described previously^23^. The ST solution was prepared by dissolving urea to a concentration of 2.0 mol·dm^−3^ in a simulated body fluid (SBF (1.5)), which is prepared like standard SBF (1.0), but at a 1.5-fold greater concentration^24^. An aqueous urease solution (0.03 cm^3^ of 0.1 mass%) was added to the ST solution (5 cm^3^), and the heated Ti pins were immersed in this solution at 50 °C for 1 day. The ST was then replaced with SBF (1.5), and the pins were kept at 50 °C for 1 week, with daily changes of the SBF (1.5). The resulting HAp-film-coated Ti (HAp-Ti) pins were then immersed in an IP6 solution (1000 ppm, 5 cm^3^) at 50 °C for 1 day, to modify the HAp film surface (HAp-IP6-Ti). They were then washed with deionized water, and subsequently immersed for 15 minutes in a silver nitrate solution at 1.00, 5.00, or 10.00 mmol·dm^−3^, to bind Ag^+^ to the film surface through IP6 chelation, thereby producing HAp-IP6-Ag^+^(1)-Ti, HAp-IP6-Ag^+^(5)-Ti, and HAp-IP6-Ag^+^(10)-Ti pins, respectively (Fig. 1e).

### Surface evaluation of the implant coated with ionic silver bound to HAp film via IP6-film chelation

The implant surface was analysed by scanning electron microscopy (SEM) after each step (Fig. 1f–j), which verified the precipitation of carbonate-containing HAp single-phase particles as a layer on the Ti substrate (Fig. 1g), and revealed the microstructures on the HAp-IP6-Ag^+^(1, 5, 10)-Ti pins (Fig. 1h–j). SEM images of HAp-IP6-Ag^+^(1) showed an agglomeration of small particles coating on the surface of the implant (Fig. 1h), indicating that the HAp layer was preserved. The images of HAp-IP6-Ag^+^(5, 10) showed an agglomeration of cube-shaped particles (arrows in Fig. 1i,j) deposited on the surface of the HAp layer. Energy dispersive X-ray (EDX) spectra showed the presence of Ag^+^ in the HAp-IP6-Ag^+^(10)-Ti pins (Fig. 1k). Thin-film X-ray diffractometry (TF-XRD) revealed the presence of the HAp phase on HAp-IP6-Ag^+^-Ti pins fabricated by immersion in solutions containing 0 to 10 mmol·dm^−3^ Ag^+^; however, it also showed that silver orthophosphate (Ag_3_PO_4_) and silver oxide (Ag_2_O) were also present on the HAp-IP6-Ag^+^(10)-Ti pins as by-products (Supplementary Fig. S1). The amount of Ag_3_PO_4_ and Ag_2_O increased with the silver nitrate concentration. Thus, the cube-like particles visible in the SEM images were Ag_3_PO_4_ or Ag_2_O crystals formed by the reaction of HAp with AgNO_3_.

**Figure 2:**
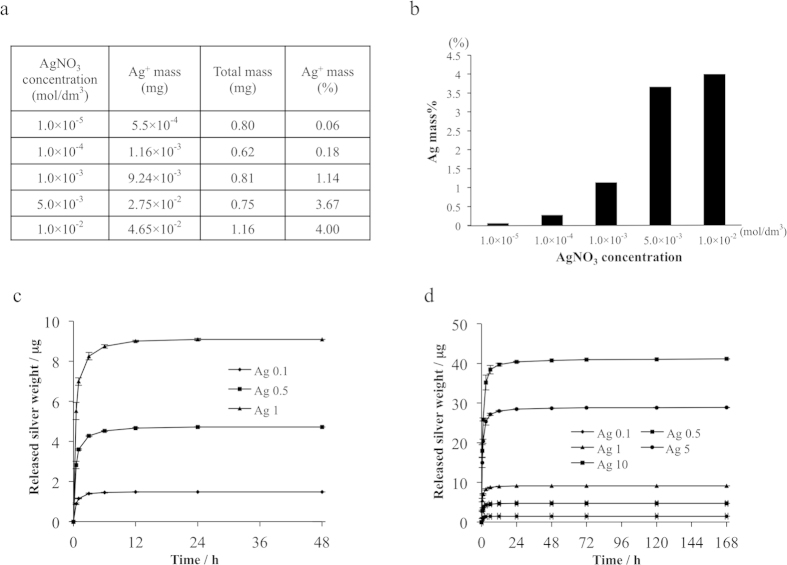
Quantification of the ionic silver fixed on the HAp film surface, and the time course of Ag^+^ ion release from the HAp-IP6-Ag^+^ coating. (**a**,**b**) The amount of ionic silver immobilized on the HAp film via IP6 chelation was measured by inductively coupled plasma atomic emission spectroscopy (ICP-AES). The silver content of the coating could be controlled in the range of 0.06 to 4.00 mass% by changing the concentration of Ag^+^ ions: solution concentrations of 0.01, 0.10, 1.00, 5.00, and 10.00 mmol·dm^−3^ produced a mass% of 0.06, 0.18, 1.14, 3.67, and 4.00, respectively. The Ag^+^ mass% = the mass of silver ions/the total mass of HAp-film coating (including the ionic silver and IP6). (**c**,**d**) Time course of cumulative Ag^+^ ion release. The samples were analysed by ICP-AES to quantify the newly released Ag^+^ ions from the HAp-IP6-Ag^+^(0.1, 0.5, 1.0, 5.0, 10.0)-Ti plates (n = 4), and the cumulative Ag^+^ ion release at each time point was calculated. The cumulative Ag^+^ ion release plateaued by 24 hours from the HAp-IP6-Ag^+^(0.1, 0.5, 1.0)-Ti plates **(c)**, and by 72 hours from the HAp-IP6-Ag^+^(5.0, 10.0)-Ti plates (**d**).

The Ag^+^ content in the implant could be measured by inductively coupled plasma atomic emission spectroscopy (ICP-AES), and was maintained in the range of 0.06 to 4.00 mass% by changing the concentration of Ag^+^ ions. Concentrations of 0.01, 0.10, 1.00, 5.00, and 10.0 mmol·dm^−3^ resulted in an Ag^+^ content of 0.06, 0.18, 1.14, 3.67, and 4.00 mass%, respectively (Fig. 2a,b).

### Time course of Ag^+^ ion release from HAp-IP6-Ag^+^ coatings

The Ag^+^ ion release from HAp-IP6-Ag^+^-Ti plates with different Ag^+^ ion concentrations (0.01, 0.10, 1.00, 5.00, and 10.0 mmol·dm^−3^) was assessed. The HAp-IP6-Ag^+^-Ti plates were immersed in 4-(2-hydroxyethyl)-1-piperazineethanesulfonic acid (HEPES) buffer (2 ml) and incubated at 37 °C. The HEPES buffer was then removed after 0.5, 1, 3, 6, 12, 24, 48, 72, 120, and 168 hours of incubation, and replaced with fresh buffer. The removed samples were subjected to ICP-AES to quantify the newly released Ag^+^ ions from the HAp-IP6-Ag^+^(0.1, 0.5, 1.0, 5.0, 10.0)-Ti plates (n = 4), and the cumulative Ag^+^ ion release at each time point was calculated. The time course of cumulative Ag^+^ ion release is shown in Fig. 2c,d. The cumulative Ag^+^ ion release plateaued by 24 hours from the HAp-IP6-Ag^+^(0.1, 0.5, 1.0)-Ti plates (Fig. 2c), and by 72 hours from the HAp-IP6-Ag^+^(5.0, 10.0)-Ti plates (Fig. 2d).

### Antibacterial effect of ionic-silver coating

#### *In vitro* study: inhibition-zone assay and cytotoxicity evaluation of the ionic silver coating

Next, we assayed the *in vitro* antibacterial effect and cytotoxicity of the Ag^+^ ions coating the substrate. Control, HAp-Ti, HAp-IP6-Ti, and HAp-IP6-Ag^+^(1, 5, 10)-Ti pins were placed on bioluminescent *S. aureus* (Xen-29) bacteria cultured in Luria Bertani (LB) medium. Each sample’s antimicrobial activity *in vitro* was assessed by the inhibition-zone method (n = 3), and the bioluminescent signal was captured as false-colour photon-count images.

**Figure 3:**
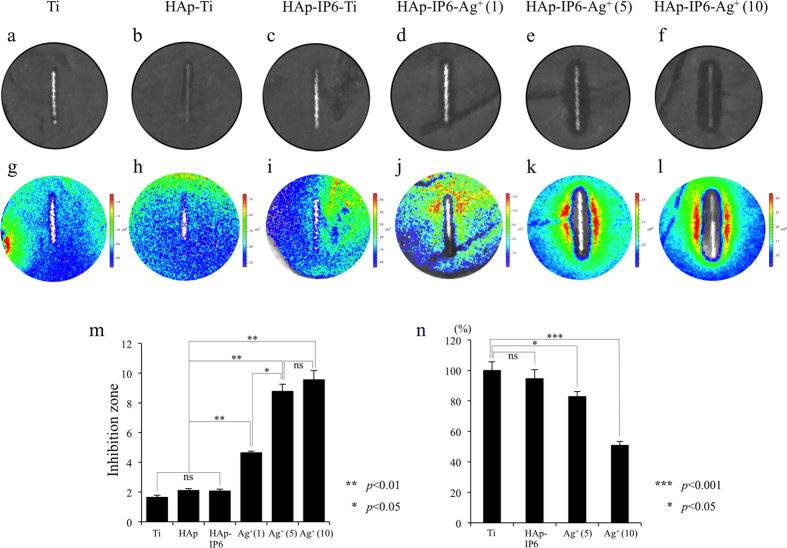
*In vitro* antibacterial effect and cytotoxicity of the bacteria-resistant HAp coating with IP6-immobilized ionic silver. Ti pins (0.5 mm diameter × 8 mm length), without surface modification (control, left) or coated with HAp, HAp-IP6, or HAp-IP6-Ag^+^ (concentrations indicated in mmol·dm^−3^), were placed on bioluminescent *S. aureus* (Xen-29). (**a–f**) Areas without living bacteria appear black. (**g–l**) Pseudo-coloured images were used to demonstrate antimicrobial activity. (**a–c**,**g–i**) No antimicrobial effects were detected with the Ti, HAp-Ti, or HAp-IP6-Ti pins. (**d–f**,**j–l**) In contrast, the HAp-IP6-Ag^+^-Ti pins showed Ag^+^ dose-dependent antimicrobial activity. (**m**) Quantified growth inhibition zones demonstrated significant bacterial growth inhibition by the HAp-IP6-Ag^+^(1, 5, 10)-Ti pins, compared to the Ti, HAp-Ti, and HAp-IP6-Ti pins *(p* <0.01 each). (**n**) L-929 cells grown in MEM conditioned by incubation with Ti, HAp-IP6-Ti, or HAp-IP6-Ag^+^(5, 10)-Ti pins. The average rate of cell rounding and cell death was 18% with the HAp-IP6-Ag^+^(5) pins and 55% with the (10)-Ti pins. Data are shown as means ± SEM.

No antimicrobial effect was detected with the Ti, HAp-Ti, or HAp-IP6-Ti pins (Fig. 3a–c,g–i). In contrast, the HAp-IP6-Ag^+^(1, 5, 10)-Ti pins showed Ag^+^ dose-dependent antimicrobial activity (Fig. 3d–f,j-l). In addition, the bacterial-growth inhibition zone of the HAp-IP6-Ag^+^(1, 5, 10)-Ti samples differed significantly from those of the Ti, HAp-Ti, or HAp-IP6-Ti pins (*p* < 0.01 each) (Fig. 3m). The inhibition zone values increased with the level of Ag^+^, and pins with at least 5.00 mmol·dm^−3^ Ag^+^ induced marked antibacterial activity.

Cytotoxicity was assayed by incubating mouse L-929 fibroblasts (ATCC CCl-1) in Minimal Essential Media (MEM) that had been conditioned by incubation with Ti, HAp-IP6-Ti, or HAp-IP6-Ag^+^(1, 5, 10)-Ti pins. The average rate of cell rounding and death was 18% for HAp-IP6-Ag^+^(5)-Ti and 55% for HAp-IP6-Ag^+^(10)-Ti pins. Cell toxicity was similar between the control and HAp-IP6-Ti pins but differed significantly between the control and HAp-IP6-Ag^+^(5)-Ti and HAp-IP6-Ag^+^(10)-Ti pins (*p* < 0.05, *p* < 0.001, respectively) (Fig. 3n). The toxicity of the HAp-IP6-Ag^+^(5)-Ti pins was less than 25%, which is acceptable according to Food and Drug Administration (FDA) standards. Together, these results indicated that the HAp-IP6-Ag^+^(5)-Ti pins were safe and suitable for *in vivo* experiments.

#### *In vivo* study: antibacterial effects of ionic-silver coating in a murine model of implant-associated osteomyelitis

We evaluated the antimicrobial efficacy of the HAp-IP6-Ag^+^(5)-Ti pin *in vivo* using a bioluminescent murine osteomyelitis model that enables infection to be monitored in real time; this model allows us to determine the efficacy of antimicrobial treatments without sacrificing the animals^25^. Our osteomyelitis model may offer an alternative pre-clinical screening tool to evaluate *in vivo* therapeutic strategies before conducting studies in larger animals and human subjects.

**Figure 4:**
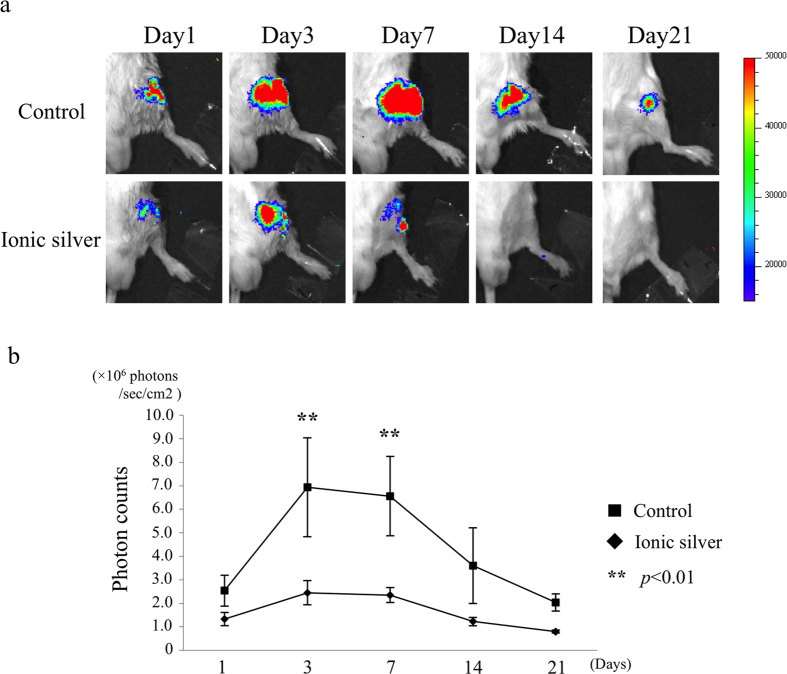
Antibacterial effect of the bacteria-resistant HAp film with IP6-immobilized ionic silver *in vivo* in a bioluminescent murine osteomyelitis model. To evaluate the antimicrobial efficacy of HAp-IP6-Ag^+^-Ti pins *in vivo*, we used a bioluminescent murine osteomyelitis model, in which osteomyelitis can be quantified and monitored throughout the course of the disease. Using this model, the efficacy of an antimicrobial coating can be demonstrated in real time. (**a**) Immediately after inoculating the femur with *S. aureus* strain (1.0 × 10^8^ CFU in 1 μl medium) and implanting a control (HAp) or ionic-silver-coated HAp-IP6-Ag^+^(5)-Ti pin into the femur, a stable luminescence signal was observed (data not shown). No luminescent bacterial signals were detected 21 days after inoculation in the group with ionic-silver-coated pins. (**b**) Sequential analysis of the bacterial luminescence revealed that the mean photon intensity (PI) was significantly lower in the ionic-silver group than in the control group 3 and 7 days after surgery. Data are shown as means ± SEM.

Mice were inoculated with a bioluminescent *S. aureus* strain (1.0 × 10^8^ CFU in 1 l medium) and received a HAp-Ti (control group) or HAp-IP6-Ag^+^(5)-Ti (ionic-silver group) implant in the same femur (n = 5, 5). Stable bioluminescence signals were observed in all of the animals immediately after inoculation. Sequential analysis of the luminescence showed a significantly lower mean bacterial photon intensity (PI) in the ionic-silver group than in the control group on postoperative days 3 and 7 (Fig. 4a,b). In the ionic-silver group, no bacterial signals were detected 21 days after inoculation, and no infection was present 3 months after surgery. These data suggest that silver ions on the HAp-IP6-Ag^+^(5)-Ti pins destroyed all of the inoculated bacteria in the femur within 21 days. In contrast, bioluminescent signals were maintained for more than 3 months in the control group (data not shown).

### Serological evaluation

**Figure 5:**
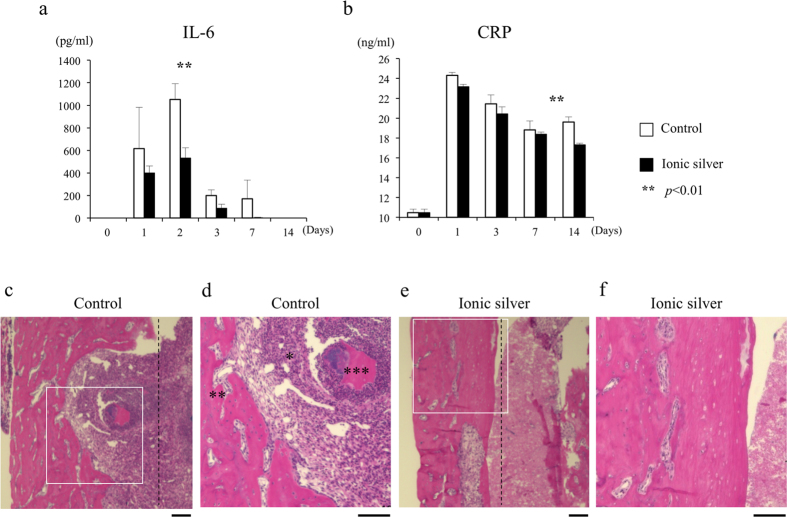
Serological and histological evaluation of the control and ionic-silver implant groups. (**a**) By post-operative day 2, the mean serum IL-6 level in the ionic-silver group was significantly lower than in the control group. (**b**) The mean serum CRP level in the ionic-silver group decreased significantly below the control group by day 14, in the subacute phase. (**c–f**) The pins were removed from the femur. (There was no apparent adhesion between the implant and the femur, because there was a small space between them in this experiment). Longitudinal femur sections from the ionic-silver and control groups were subjected to hematoxylin and eosin staining on day 28 after bacterial inoculation. (**d**,**f**) Magnified views of the boxes in (**c,e**), respectively. Control-group samples showed bacterial colonies in the medullary cavity of the femur, with marked neutrophil infiltration (*). Manifestations of chronic osteomyelitis, such as new bone formation with trabecular bone resorption by osteoclasts (**) and sequestrum (***), were markedly less frequent in the ionic-silver group. The black dotted line indicates the location of the border of the HAp-Ti pin (control) or HAp-IP6-Ag^+^(5)-Ti pin (ionic silver) before its removal. Data are shown as means ± SEM. Bars = 100 μm.

To evaluate the infection in the staphylococcal osteomyelitis model, we measured the serum interleukin-6 (IL-6) and C-reactive protein (CRP) in retro-orbital blood samples collected before surgery and on post-surgical days 1, 3, 7, and 14 from mice in the control and ionic-silver groups (n = 3, 3). In this mouse model, IL-6 is elevated in the serum and infected bone in the early post-infection period^26^. In addition, CRP is a valuable clinical marker for infectious processes. In the present study, the mean serum IL-6 was elevated in the acute phase, but it was significantly lower in the ionic-silver group than the control group on day 2 (Fig. 5a). CRP was initially elevated in both groups in the acute phase, probably in response to the surgery. The mean serum CRP in the ionic-silver group dropped significantly below that in the control group by day 14, during the subacute phase; CRP levels remained elevated longer in the control group (Fig. 5b). These results were consistent with the bioluminescence observations.

### Histological analysis

Histological analysis of femur specimens collected on post-operative day 28 revealed bacterial colonies in the medullary cavity of the femur, with marked neutrophil infiltration, in the control group. Obvious new bone formation with trabecular bone resorption by osteoclasts and sequestrum were also frequent in the control group. These manifestations of chronic osteomyelitis were markedly less frequent in the ionic-silver group (Fig. 5c–f). Note that because there was a small space between the implant and the femur in this experiment, there was no apparent adhesion or bone conduction.

## Discussion

Our novel bacteria-resistant HAp film forms a stable, uniformly dense coating of ionic silver immobilized by chelation with an IP6 film. To our knowledge, this is the first report showing that an ionic-silver chelation coating on a metal implant had significant and reproducible antibacterial effects both *in vitro* and *in vivo*. Our coating has several advantages: 1) it retains significant antibacterial activity even *in vivo* because of the strong ionic-silver chelation; 2) the precipitation process creates a uniform silver coating, even on complex structures; and 3) the immersion process does not require high temperatures, making it applicable to metals, plastics, or ceramics. Although several silver-containing devices have demonstrated an antimicrobial effect on surrounding tissues, these devices are typically produced using high-temperature processes, such as the plasma spray method^11^, that heat the device to several thousand degrees Celsius. These methods are not practical for many materials, including plastics and ceramics. Our ionic-silver chelating technique requires much lower temperatures, but still binds silver ions strongly by IP6 chelation.

Previous studies showed that silver has good biocompatibility without cytotoxicity^27^; however, toxicity is still a concern because silver’s effects are dose-dependent. One study found that silver became toxic to cultured fibroblasts at a local concentration of 1200 ppb^28^. Devices with a high local concentration of silver cannot be used medically. The most common complication of high silver exposure is argyria, a grey-blue discoloration of the tissues, and systemic argyrosis results from 4–6 g silver^29^. With our coating, the total silver in a 0.5 mm × 8 mm pin is about 0.457 mg. For humans, the total silver in a femur-nailing implant (20 mm × 300 mm) coated by our method would be 685 mg. Therefore, our ionic-silver coating provides a low total amount of silver. This coating also showed less than 25% cytotoxicity when produced with a silver ion concentration less than 5.00 mmol·dm^–3^, which is within the FDA’s acceptable range. Thus, neither cytotoxic effects nor systemic argyrosis are likely to be a problem with our coating technique. Taken together, our findings suggest that this novel coating can safely provide significant clinical protection against infections associated with metal or plastic implants.

In conclusion, we developed a novel bacteria-resistant hydroxyapatite film in which ionic silver is immobilized via inositol hexaphosphate chelation using a low-heat immersion process. This coating demonstrated significant antibacterial activities both *in vitro* and *in vivo*. Because this coating is produced by a low-heat immersion process, it can be applied to complex structures of various materials, to provide significant protection against implant-associated infections.

## Methods

### Surface evaluation of the implant coated with ionic silver bound to HAp film via IP6-film chelation

To characterize our novel bacteria-resistant implants, we used XRD (Rigaku, Japan) and SEM (JEOL, Japan) for phase identification and morphological observations, respectively. We used an EDX apparatus attached to the SEM to detect the presence of silver, and used ICP-AES (SII, Japan) to quantify silver ions in the implants. The crystalline phases of the resulting powders were identified using TF-XRD (SmartLab, Rigaku Co., Japan) generating CuKα radiation at 45 kV and 200 mA. Data were collected in the range 2θ = 20–50° with a step size of 0.02° and a scan speed of 1 degree/min (incident angle: 0.1°). The crystalline phase was identified using JCPDS reference patterns for HAp (#09–0432).

### Measurement of Ag^+^ ion release from the HAp-IP6-Ag^+^ coating

The HAp-IP6-Ag^+^ coating was applied to Ti plates (Tanaka Medical Instruments Co, Ltd., Tokyo, Japan) at 0.01, 0.10, 1.00, 5.00, and 10.0 mmol·dm^−3^, then the Ag^+^ ion release from the HAp-IP6-Ag^+^-Ti plates was assessed. The HAp-IP6-Ag^+^-Ti plates were immersed in 20 mmol/dm^3^ HEPES buffer (pH 7.4; 2 ml) in separate wells, and incubated at 37 °C with agitation. The HEPES buffer was removed after 0.5, 1, 3, 6, 12, 24, 48, 72, 120, 168 hours of incubation, and replaced with new HEPES buffer. Samples of the removed buffer (1.8 cm^3^) were diluted with a standard solution (0.5 ppm, 0.5 cm^3^), and 2.2 cm^3^ of ultrapure water. ICP-AES was used to quantify the Ag^+^ ions newly released from the HAp-IP6-Ag^+^(0.1, 0.5, 1.0, 5.0, 10.0)-Ti plates (n = 4), and the cumulative level of released Ag^+^ ions at each time point was calculated.

### Bioluminescent bacteria

We used Xen-29, a bioluminescent strain of *S. aureus*, for bacterial experiments. Xen-29 was obtained from Caliper LS Co. (Hopkinton, MA). The bacteria were cultured in LB (Sigma-Aldrich Co., St. Louis, MO) at 37 °C under ambient aeration with gentle agitation, and was selectively grown on medium containing 200 μg/ml kanamycin, as previously reported^25^. *S. aureus* Xen-29, derived from the parental American Type Culture Collection (ATCC) 12600 strain, has a stable copy of a modified *Photorhabdus luminescens luxABCDE* operon encoding the enzymes responsible for the luminescent reaction. Since bacterial bioluminescence requires no additional substrate, the organism constitutively emits a bioluminescent signal as long as it is viable. Bacteria samples were frozen and stored at −80 °C, and then thawed at 4 °C for one hour prior to each experiment. Typically, bacterial viability was maintained at 4 °C for approximately 5 hours after thawing.

### Bioluminescence imaging

Bioluminescence was observed with a Caliper LS-IVIS^®^ Lumina (Summit Pharmaceuticals International Co., Tokyo, Japan) cooled CCD optical macroscopic imaging system. The photon emissions of the bacterial bioluminescent signal were captured as false-colour photon-count images and quantified with Living Image software version 3.0 (Caliper LS Co., Hopkinton, MA). The bacterial PI was expressed as photon flux, in units of photons/sec/cm^2^/steradian. To quantify the bacterial PI, the range of interest (ROI) was defined over the bacteria-plating area and examined with the same ROI.

### Antibacterial effect of ionic-silver coating

#### *In vitro* study: inhibition-zone assay

The bacterial inhibition zone was used to quantify antibacterial activity resulting from the diffusion of the antibacterial agent through an agar medium. A thin agar plate was poured, and Xen-29 *S. aureus* was distributed on the plate. An unmodified (control) Ti pin or an HAp-Ti, HAp-IP6-Ti, or HAp-IP6-Ag^+^(1, 5, 10)-Ti pin was then placed over the agar layer at a pre-determined position, and the plates were incubated at 37 °C for 24 hours. The inhibition zone was calculated using Equation (1), where D_1_ and D_2_ were the area of the inhibition zone and the testing pin, respectively:


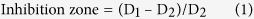


We also evaluated the antimicrobial activity using a bioluminescence imaging system. Bioluminescent bacterial signals surrounding the Ti pins were captured by a Caliper LS-IVIS^®^ Lumina.

#### *In vitro* evaluation of the cytotoxicity of the ionic silver coating

To assess cell toxicity, we prepared Ti pins (0.5 mm diameter × 8 mm length) without surface modification (control), and HAp-IP6-Ti, and HAp-IP6-Ag^+^(1, 5, 10)-Ti pins. Following the ISO standard protocol 10993-5, each pin was incubated in a separate well in MEM (Sigma-Aldrich Co., St. Louis, MO) with 5% bovine serum at a concentration of 3 cm^2^/ml for 24 hours at 37 ^o^C with agitation. Mouse fibroblast L-929 cells (ATCC CCl-1) were seeded in the wells of cell-culture plates and incubated until approximately 80% confluent. The cell-culture medium was then replaced by the unfiltered extract medium and the cells were incubated at 37 ^o^C in 5% CO_2_ for 3 days (n = 3). The condition of the cells in each well was then assessed.

#### *In vivo* study: bioluminescent murine osteomyelitis model

We used a bioluminescent murine osteomyelitis model to evaluate the antimicrobial activity of HAp-IP6-Ag^+^(5)-coated pins *in vivo*^25^. We used 16 adult male BALB/c mice (12 weeks old, 20–25 g weight) purchased from Sankyo Labo Service (Shizuoka, Japan). The mice were maintained in our animal facility under specific pathogen-free conditions. For the surgery, mice were anesthetized with an intraperitoneal injection of 50 mg/kg pentobarbital, and the skin on the left hind knee was shaved and sterilized with povidone iodine. A skin incision was made over the knee, and the distal femur was exposed through a lateral parapatellar arthrotomy with medial displacement of the quadriceps-patellar complex. The distal end of the femur was perforated using a high-speed drill with a 0.5-mm sharp steel burr (Fine Science Tools Inc., Canada). A channel was created using a 23G (external diameter, 0.6 mm) needle, and a bioluminescent strain of *S. aureus* (1.0 × 10^8^ CFU in 1 μl medium) was injected into the medullary cavity of the femur using a Hamilton syringe. Next, either a HAp-coated (control) or HAp-IP6-Ag^+^ titanium pin was inserted into the medullary cavity (n = 5, 5) and the burr hole was closed with bone wax. The quadriceps-patellar complex was reduced, and the muscle and skin openings were closed with sutures. The animals were placed on a heating pad and monitored closely until they displayed spontaneous forelimb movement and began drinking water.

Bacterial PI was sequentially measured with a Caliper LS-IVIS^®^ Lumina at various time points; each mouse was anesthetized via inhaled aerosolized isoflurane mixed with oxygen, placed on its back, and imaged for 5 minutes. All of the experiments were approved by the Animal Care and Use Committee of Keio University. And, these experiments were carried out in accordance with the approved guidelines.

### Serological evaluation

Blood samples were collected by retro-orbital bleeding before surgery and on days 1, 3, 7, and 14 after surgery. Serum IL-6 and CRP were measured by ELISA (R&D Systems and Kamiya Biomedical Company, respectively) according to the manufacturers’ instructions.

### Histological analysis

On day 28 after the operation, mice in both groups were sacrificed. The femurs were removed and separated from soft tissues. Because a channel was created in the femur using a 23G (external diameter, 0.6 mm) needle before the *S. aureus* inoculation and implant insertion into the femur, there was a small space between the implant (external diameter, 0.5 mm) and the femur. Although we frequently observed bone ingrowth into the surface of the Ti implant in non-infected animals (data not shown), no apparent adhesion between the implant and the femur was detected in the current infection models, so the pins could be removed from the femur smoothly and gently. The femur samples were fixed in 4% paraformaldehyde, demineralized with ethylenediaminetetraacetic acid, embedded in paraffin, and sectioned at 5 m. Specimens were stained with hematoxylin and eosin.

### Statistical analysis

Differences between the bacterial-growth inhibition zone of Ti, HAp-Ti, HAp-IP6-Ti, and HAp-IP6-Ag^+^(1, 5, 10)-Ti pins; and differences in bacterial PI, serum IL-6, and CRP levels in the control and ionic-silver groups were statistically analysed by one-way ANOVA and the Fisher post-hoc test. A probability value of less than 0.05 was considered significant in all statistical analyses. All data are expressed as the mean ± standard error.

## Additional Information

**How to cite this article**: Funao, H. *et al*. A novel hydroxyapatite film coated with ionic silver via inositol hexaphosphate chelation prevents implant-associated infection. *Sci. Rep*. **6**, 23238; doi: 10.1038/srep23238 (2016).

## Supplementary Material

Supplementary Information
