# Supplementary material for: A novel hydroxyapatite film coated with ionic silver via inositol hexaphosphate chelation prevents implant-associated infection
Source: Sci Rep. 2016 Mar 17;6:23238. doi: 10.1038/srep23238 (PMC4794646; doi:10.1038/srep23238)
Supplement: Supplementary Information [file srep23238-s1.pdf]

Title:

A novel hydroxyapatite film coated with ionic silver via inositol hexaphosphate chelation prevents implant-associated infection

Authors:

Haruki Funao, M.D., Ph. D.[1], Shigenori Nagai, Ph. D.[2,5], Aya Sasaki, M.D., Ph. D.[3], Tomoyuki Hoshikawa[4], Takashi Tsuji, M.D., Ph. D.[1], Yasunori Okada, M.D., Ph. D.[3,6], Shigeo Koyasu, M.D., Ph. D.[2,7], Yoshiaki Toyama, M.D., Ph. D.[1], Masaya Nakamura, M.D., Ph. D.[1], Mamoru Aizawa, Ph. D.[4], Morio Matsumoto, M.D., Ph. D.[1], Ken Ishii, M.D., Ph. D.[1]

Affiliation:

[1]Department of Orthopaedic Surgery, [2]Department of Microbiology and Immunology, [3]Department of Pathology, Keio University School of Medicine, Shinjuku, Tokyo, JAPAN

[4]Department of Applied Chemistry, School of Science and Technology, Meiji University, Kawasaki, Kanagawa, JAPAN

[5]Department of Molecular Immunology, Graduate School of Medical and Dental Sciences, Tokyo Medical and Dental University, Bunkyo, Tokyo, JAPAN

[6]Department of Pathology and Oncology, School of Medicine, Juntendo University, Bunkyo, Tokyo, JAPAN

[7]Laboratory for Immune Cell Systems, RIKEN Centre for Integrative Medical Sciences (IMS), Yokohama, Kanagawa, JAPAN

Corresponding author: Ken Ishii, M.D., Ph. D., Department of Orthopaedic Surgery, Keio University School of Medicine, Address: 35 Shinjuku, Tokyo, 160-8582, JAPAN Phone: +81-3-5363-3812 Fax: +81-3-3353-6597, Email: keni8888@z7.keio.jp

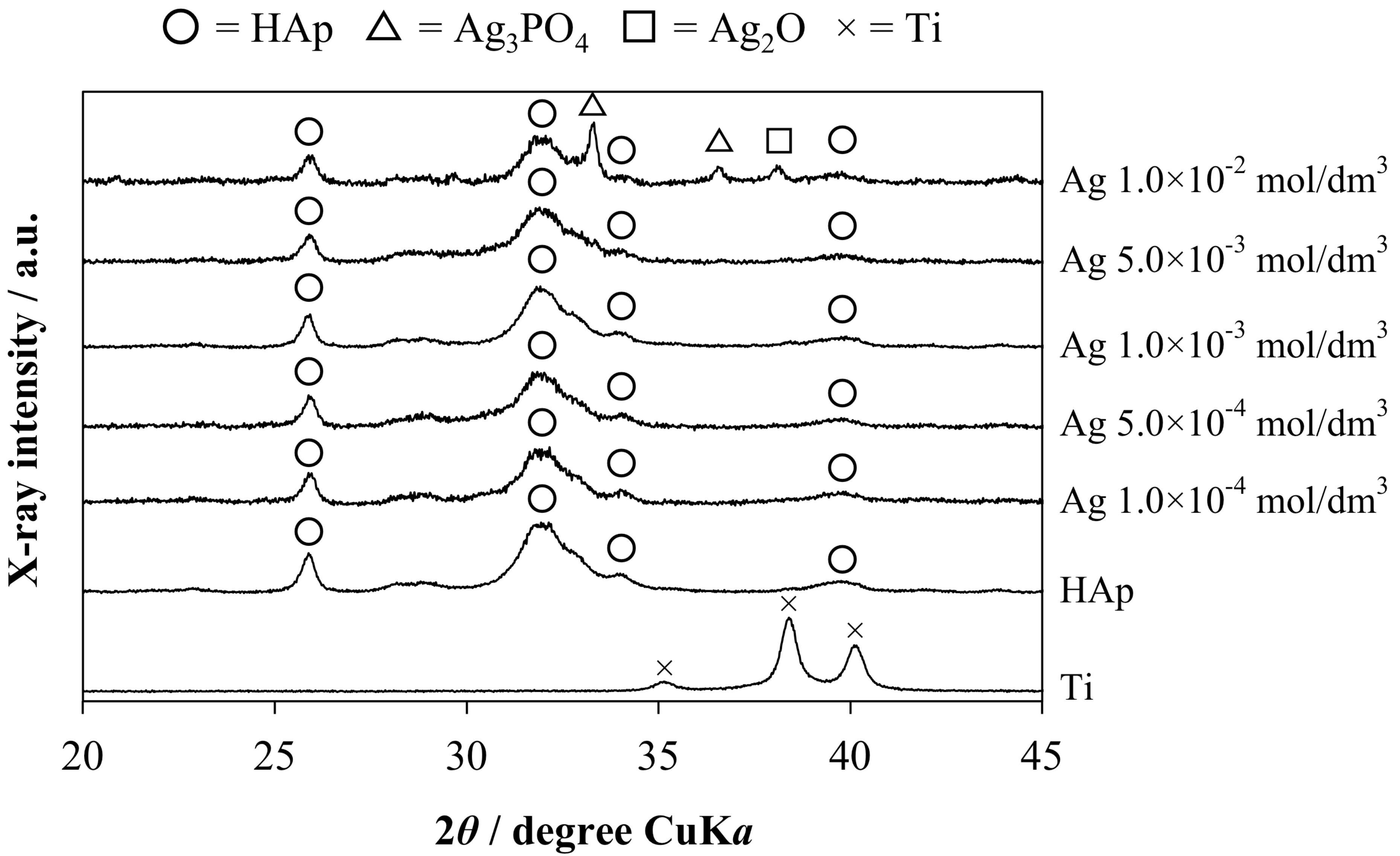
○ = HAp △ = Ag3PO4 □ = Ag2O × = Ti
Ag 1.0×10-2 mol/dm3
Ag 5.0×10-3 mol/dm3
Ag 1.0×10-3 mol/dm3
Ag 5.0×10-4 mol/dm3
Ag 1.0×10-4 mol/dm3
HAp
Ti
X-ray intensity / a.u.
20
25
30
35
40
45
2θ / degree CuKa

Supplementary Figure S1. Thin-film X-ray diffractometry (TF-XRD) analysis of the bacteria-resistant HAp coating with IP6-immobilized ionic silver

TF-XRD showed the presence of the HAp phase on HAp-IP6-Ag^+-Ti pins fabricated by immersion in solutions with 0 to 10 mmol· dm^{-3} Ag^+; however, silver orthophosphate (Ag_3PO_4) and silver oxide (Ag_2O) were also present on the HAp-IP6-Ag^+(10)-Ti pins as by-products. The amount of Ag_3PO_4 and Ag_2O increased with the silver nitrate concentration. The measurement conditions were as follows; target material: Cu, voltage: 45 kV, current: 200 mA, incident angle: 0.1 degree, scan speed: 1.0000 degree/min, step size: 0.02 degree, scan axis: 2 θ, scan range: 20.000 - 50.000 degrees.
